# Characteristics of Adults Seeking Health Care Provider Support Facilitated by Mobile Technology: Secondary Data Analysis

**DOI:** 10.2196/humanfactors.8246

**Published:** 2017-12-21

**Authors:** Kelly Bosak, Shin Hye Park

**Affiliations:** ^1^ School of Nursing University of Kansas Medical Center Kansas City, KS United States

**Keywords:** health technology, health behavior, health care provider, cohort analysis

## Abstract

**Background:**

Mobile health technology is rapidly evolving with the potential to transform health care. Self-management of health facilitated by mobile technology can maximize long-term health trajectories of adults. Little is known about the characteristics of adults seeking Web-based support from health care providers facilitated by mobile technology.

**Objective:**

This study aimed to examine the following: (1) the characteristics of adults who seek human support from health care providers for health concerns using mobile technology rather than from family members and friends or others with similar health conditions and (2) the use of mobile health technology among adults with chronic health conditions. Findings of this study were interpreted in the context of the Efficiency Model of Support.

**Methods:**

We first described characteristics of adults seeking Web-based support from health care providers. Using chi-square tests for categorical variables and *t* test for the continuous variable of age, we compared adults seeking Web-based and conventional support by demographics. The primary aim was analyzed using multivariate logistic regression to examine whether chronic health conditions and demographic factors (eg, sex, income, employment status, race, ethnicity, education, and age) were associated with seeking Web-based support from health care providers.

**Results:**

The sample included adults (N=1453), the majority of whom were female 57.60% (837/1453), white 75.02% (1090/1453), and non-Hispanic 89.13% (1295/1453). The age of the participants ranged from 18 to 92 years (mean 48.6, standard deviation [SD] 16.8). The majority 76.05% (1105/1453) of participants reported college or higher level of education. A disparity was found in access to health care providers via mobile technology based on socioeconomic status. Adults with annual income of US $30,000 to US $100,000 were 1.72 times more likely to use Web-based methods to contact a health care provider, and adults with an annual income above US $100,000 were 2.41 to 2.46 times more likely to access health care provider support on the Web, compared with those with an annual income below US $30,000. After adjusting for other demographic covariates and chronic conditions, age was not a significant factor in Web-based support seeking.

**Conclusions:**

In this study, the likelihood of seeking Web-based support increased when adults had any or multiple chronic health conditions. A higher level of income and education than the general population was found to be related to the use of mobile health technology among adults in this survey. Future study is needed to better understand the disparity in Web-based support seeking for health issues and the clinicians’ role in promoting access to and use of mobile health technology.

## Introduction

### Mobile Health Technology

Mobile health technology is rapidly evolving with the potential to transform health care. Mobile devices, including mobile phones, tablet computers, and handheld devices with wireless Internet connectivity offer new opportunities to maximize health and wellness and improve long-term health trajectories for adults across the age continuum [1]. Mobile technology has been used to promote health behaviors, such as physical activity [2] and optimal nutrition [3], major determinants of health [4] that enhance physical and mental function [5] for healthy aging.

Self-management of health, defined as individuals assuming tasks to deal with medical management, role management, or emotional aspects of health conditions [6], is facilitated by mobile technology. Optimizing self-management of health is associated with an increase in average life years, a delay in the development of chronic health conditions, and lower Medicare costs [7]. Despite the advantages, the actual reach and availability of coordinated activities and programs on the Internet designed to promote self-management using mobile technology have been lower than expected, and attrition rates were high [8]. Individual engagement in positive health behaviors is necessary to achieve the desired outcomes [9]. Integrating health care provider support with this technology may be the key to improving this process.

### Study Aim

The aim of this study was to assess the characteristics of adults across the lifespan, who seek health care support from providers facilitated by mobile technology. We selected an existing dataset to investigate Web-based human support seeking from providers rather than from family members and friends or others with similar health conditions. The impact of sociodemographic factors and other variables on Web-based support seeking for health issues from providers remains unclear. Understanding the characteristics of mobile technology users who do and do not seek Web-based support for health care issues from providers is needed for translation to clinical practice and to inform future health behavior research.

### Background and Significance

The impact of mobile technology in the daily lives of people worldwide has increased markedly over the past decade and continues to expand. Mobile technology offers distinct advantages for optimizing health and wellness, with unlimited reach across economic and geographic boundaries, as well as continuous availability. Most Americans (95%) now own a mobile phone of some kind [10]. Furthermore, over half (62%) of mobile phone owners use their phone to search for health information [11]. Web-based searching for health information facilitated by mobile technology differs based on sociodemographic profile [12]. Mobile phone owners who were Latino or African American, aged between 18 and 49 years, and had a college degree were more likely to access Web-based health information [11]. Although the majority of mobile technology users report searching for Web-based health information, less is known about the human element in Web-based communication and information sharing. The characteristics of individuals who use mobile technology to seek health care support from providers remain unclear. Greater understanding of Web-based health care support seeking from providers is needed to inform best practices for retaining individuals in positive health behaviors over time.

This investigation builds on existing data. A study using Pew Research Center survey data assessed the potential reach of mobile phones among adults and found that chronic health conditions affected mobile technology use. Slightly less than one quarter of the sample had diabetes, and these individuals were less likely to use mobile phones [13] compared with those without diabetes. Individuals with diabetes, with higher income, younger age, and Web-based health information searching were associated with higher mobile phone use [13]. These findings are consistent with other study results that found less affluent adults with chronic health conditions were largely disconnected from the world of technological tools and services, both physically and psychologically [14]. Thus, adults with chronic conditions were less likely to use mobile phones, and this appears to be because of older age and lower socioeconomic status, whereas adults with a higher level of education, younger age, nonwhite race, and high income were more likely to use mobile phones.

A previous study also indicated that two subgroups had a lower chance to be engaged users of health care resources facilitated by mobile technology, namely, males (odds ratio [OR] 2.24, 95% CI 1.23-4.08) and younger adults (OR 1.02, 95% CI 1.00-1.04), who were also less likely to return to health care resources on the Web for follow-up after 1 week [15]. Previous studies generally assessed mobile phone use rather than Web-based health care support seeking from providers. On the basis of these findings, we predicted that females, older and more affluent adults, and adults without chronic conditions would be most likely to use mobile technology to seek Web-based health care provider support.

The Efficiency Model of Support described by Schueller et al (2016) provides a framework for understanding the provision of human support in the context of behavioral interventions facilitated by technology. This model predicts that health care provider interaction in conjunction with mobile technology leads to more frequent and more effective use (eg, with greater individual engagement and lower attrition) [16]. Health may be enhanced when data generated by mobile devices are combined with assessment and intervention from health care providers. This model frames mobile technology as the facilitator rather than the driver of positive health behavior [17]. Integrating health care provider support for individual self-management of health facilitated by mobile technology is advantageous, and this model will guide future research and clinical translation.

### Health Care Provider Perceptions of Mobile Health Technology

The health care provider perspective was not assessed in this survey; however, the perceptions of health care providers of mobile health technology use and the integration of human support must be considered to promote optimal use of the technology. A previous survey of health care professionals (n=500) found that the majority (86%) of respondents were accepting of mobile health resources and indicated that mobile technology will increase their knowledge of a patient’s condition and improve their relationships with patients [18]. This survey found that only a small number (16%) of health care professionals currently recommend mobile health resources for patients, but just less than half (46%) plan to do so in the next 5 years [18]. In addition, a survey of nonclinician decision leaders in health care (n=900), representing medical technology companies, insurance, and other stakeholder groups, found more than half indicated that wireless, wearable health tracking devices, and other health technology advancements will help improve health care delivery [19]. This study investigated the sociodemographic profile and chronic health conditions of participants related to health care support seeking from a provider using mobile technology.

## Methods

### Design and Data Collection

The design of this study was a secondary analysis of cross-sectional survey data collected by random digit dialing by Princeton Survey Research Associates International for the Pew Research Center’s Internet and American Life Project [20]. The data were collected from August to September 2012 with multiple attempts made to contact each sampled telephone number. Calls were staggered over times of day and days of the week to maximize the chance of making contact with potential respondents, and each phone number received at least one daytime call. A nationally representative sample of adults aged 18 years and older was recruited from all geographic census regions of the United States, including Northeast, Midwest, South, and West, as well as urban, suburban, and rural locations. Interviews were conducted in both English and Spanish. Telephone interviews were conducted by random digit dialing and included some individuals without a landline phone. According to Pew, within strata, phone numbers were drawn with equal probabilities [20]. Permission was obtained from the Pew Research Center to analyze the data, and the dataset was downloaded free of charge. The dataset did not contain any participant identifiers, and thus, was approved by the institutional review board at the authors’ university for an “exempt” study before beginning analyses.

### Measures

The sociodemographic profile of participants related to health care support seeking from a provider using mobile technology was investigated. Health care support seeking from a provider was defined as Web-based communication with a doctor or other health care provider (included Web-based or combination of Web-based and conventional support) the last time the respondent had a health issue. Response options also included Web-based health information seeking from friends and family members or others with a similar health condition. Covariates included chronic health conditions, defined as diagnosed conditions, such as diabetes, high blood pressure, lung conditions (asthma, bronchitis, or emphysema), heart disease (heart failure or heart attack), cancer, or any other chronic health condition.

### Data Analysis

We first described the characteristics of adults seeking support from health care providers using Web-based or conventional methods. Using chi-square test for categorical variables and *t* test for the continuous variable of age, we compared adults seeking Web-based support by demographics. The primary aim was analyzed using multivariate logistic regression to examine whether chronic health conditions and demographic factors (eg, sex, income, employment status, race, ethnicity, education, and age) were associated with seeking Web-based support from health care providers for adults.

We performed three regression analyses. Model 1 included demographic variables. Model 2 added the variable of any chronic health condition into Model 1. Model 3 included three chronic condition groups (adults with one chronic condition, those with multiple chronic conditions, and those without any chronic condition) in addition to demographics. Wald χ^2^ was reported to evaluate the fit for each regression model. All data were analyzed using the STATA version 14.0 (StataCorp, LP, College Station).

## Results

### Study Sample

The sample (Table 1) included adults (n=1453), the majority of whom were female 57.60% (837/1453), white 75.02% (1090/1453), and non-Hispanic 89.13% (1295/1453). The age of the participants ranged from 18 to 92 years (mean 48.6, SD 16.8). The majority 76.05% (1105/1453) of participants reported college or higher level of education. Of note, the category of less than or incomplete high school had few cases and was combined with the high school category. About half of the participants 50.72% (737/1453) reported any chronic health condition(s), with slightly more reporting one chronic condition than those reporting multiple chronic conditions 29.46% (428/1453) vs 21.27% (309/1453). Over half 54.16% (787/1453) of the respondents reported an income in the mid-range, and were employed 59.67% (867/1453) either part time or full time, or were self-employed. Less than one quarter 20.65% (300/1453) were retired.

### Health Care Support Seeking

Most respondents reported seeking support for a health issue from a health care provider by visiting them in person or talking on landline phone 85.07% (1236/1453), compared with Web-based support seeking 14.93% (217/1453) through the Internet or email, or a combination of Web-based and conventional methods. According to bivariate analysis (Table 2) Web-based support seeking significantly differed by income, employment, race, education, and age (*P*<.05). Adults with income above US $30,000, being employed, having achieved college or higher education, and of white race sought support from a health care provider for a health issue on the Web, rather than by conventional means. Furthermore, adults seeking Web-based support were significantly younger (mean 46.1, SD 14.7) than those seeking conventional support (mean 49.1, SD=17.1).

**Table 1:** A descriptive summary of study sample (n=1453).

Variable			Value
**Health care provider support seeking, n (%)**		
	Yes, Web-based or both Web-based and conventional means		217 (14.93)
	Conventional means only		1236 (85.07)
**Chronic condition, n (%)**			
	No		716 (49.28)
	**Yes (any chronic condition), n (%)**		737 (50.72)
		One chronic health condition	428 (29.46)
		Multiple chronic conditions	309 (21.27)
**Sex, n (%)**		
	Male		616 (42.40)
	Female		837 (57.60)
**Income, n (%)**		
	<US $30,000		353 (24.29)
	US $30,000-$100,000		787 (54.16)
	>US $100,000		313 (21.54)
**Employment, n (%)**		
	Employed (full-time, part-time, or self-employed)		867 (59.67)
	Retired		300 (20.65)
	Not employed for pay		286 (19.68)
**Race, n (%)**		
	White		1090 (75.02)
	Black or African-American		235 (16.17)
	Asian or Pacific Islander		36 (2.48)
	Other		92 (6.33)
**Ethnicity, n (%)**		
	Hispanic		158 (10.87)
	Not Hispanic		1295 (89.13)
**Education, n (%)**			
	Less than or high School		348 (23.95)
	College or higher		1105 (76.05)
Age, mean (SD)			48.63 (16.81)

**Table 2:** Comparison of Web-based and conventional support seeking (n=1453).

Variable		Web-based support (n=217) n (%)	Conventional support (n=1236) n (%)	Chi-square values (degrees of freedom)
**Chronic condition**				
	No	101 (46.54)	615 (49.76)	
	Yes (at least one chronic condition)	116 (53.46)	621 (50.24)	
**Chronic condition**				
	No	101 (46.54)	615 (49.76)	
	One chronic condition	70 (32.26)	358 (28.96)	
	Multiple chronic conditions	46 (21.2)	263 (21.28)	
**Sex**				
	Male	92 (42.4)	524 (42.39)	
	Female	125 (57.6)	712 (57.61)	
**Income**				15.3^a^ (2)
	<US $30,000	35 (16.13)	318 (25.73)	
	US $30,000-100,000	117 (53.92)	670 (54.21)	
	>US $100,000	65 (29.95)	248 (20.06)	
**Employment**				17.0^a^ (2)
	Employed (full-time, part-time, or self-employed)	152 (70.05)	715 (57.85)	
	Retired	23 (10.6)	277 (22.41)	
	Not employed for pay	42 (19.35)	244 (19.74)	
**Race**				9.4^a^ (3)
	White	148 (68.2)	942 (76.21)	
	Black or African-American	39 (17.97)	196 (15.86)	
	Asian or Pacific Islander	8 (3.69)	28 (2.27)	
	Other	22 (10.14)	70 (5.66)	
**Ethnicity**				
	Hispanic	23 (10.6)	135 (10.92)	
	Not Hispanic	194 (89.4)	1101 (89.08)	
**Education**				6.6^a^ (1)
	Less than or high school	37 (17.05)	311 (25.16)	
	College or higher	180 (82.95)	925 (74.84)	
Age, mean (SD)		46.10 (14.71)	49.07 (17.12)	2.4^a,b^

^a^*P*<.05.

^b^*t* statistic calculated from *t* test.

The multivariate logistic regression analysis of Web-based support from health care providers on chronic conditions and demographics is shown in Table 3. The odds of Web-based support seeking from a health care provider for a health condition were 1.62 times greater for adults with any chronic condition compared with adults without any chronic condition (Model 2). Furthermore, Model 3 showed that the odds of Web-based support seeking from a health care provider were 1.85 times greater when adults had multiple chronic conditions than those without multiple chronic conditions. Participants reporting other race were 2.15 times more likely to seek health support on the Web than whites. In Models 1 to 3, there were no significant differences in the odds of seeking support from a health care provider on the Web based on sex, age, or ethnicity, compared with those individuals who do not seek health information on the Web, after adjusting for covariates.

A disparity was found in access to health care providers via mobile technology based on socioeconomic status. Adults with annual income ranging from US $30,000 to US $100,000 were 1.72 times more likely to contact a health care provider on the Web, and adults with an annual income above US $100,000 were 2.41 to 2.46 times more likely to access a health care provider on the Web, compared with those with an annual income below US $30,000 (Models 2 and 3). In addition, the odds of seeking Web-based support were 1.51 to 1.53 times greater for adults with college or higher education than those with less than or high school education. In comparison with employed adults, retired adults were 57% to 58% less likely to contact a health care provider on the Web for support for a health issue, even after controlling for chronic conditions and other demographic differences, including age and income differences.

**Table 3:** Multivariate logistic regression of Web-based support from health care providers on chronic conditions and demographics (n=1453).

Web-based support		Model 1		Model 2		Model 3	
		OR^a^ (95% CI)	*P* value	OR (95% CI)	*P* value		OR (95% CI)	*P* value
**Chronic condition**								
	No (Ref.)							
	Yes			1.62 (1.17-2.23)	.003			
**Chronic condition**								
	No (Ref.)							
	One chronic condition						1.52 (1.07-2.16)	.02
	Multiple chronic conditions						1.85 (1.20-2.85)	.005
**Income**								
	<US $30,000 (Ref.)							
	US $30,000-100,000	1.65 (1.07-2.54)	.024	1.72 (1.11-2.65)	.015		1.72 (1.12-2.66)	.01
	>US $100,000	2.27 (1.38-3.72)	.001	2.41 (1.47-3.97)	.001		2.46 (1.49-4.06)	<.001
**Employment**								
	Employed (Ref.)							
	Retired	0.50 (0.27-0.79)	.004	0.43 (0.25-0.74)	.002		0.42 (0.25-0.73)	.002
	Not employed for pay	1.04 (0.70-1.54)	.86	0.97 (0.65-1.45)	.89		0.96 (0.64-1.44)	.85
**Race**								
	White (Ref.)							
	Black or African-American	1.42 (0.96-2.12)	.08	1.42 (0.95-2.11)	.08		1.40 (0.94-2.09)	.10
	Asian or Pacific Islander	1.59 (0.70-3.62)	.27	1.66 (0.73-3.78)	.23		1.67 (0.73-3.80)	.22
	Other	2.14 (1.23-3.74)	.007	2.15 (1.23-3.76)	.007		2.15 (1.23-3.77)	.007
**Ethnicity**								
	Hispanic (Ref.)							
	Not Hispanic	1.22 (0.73-2.04)	.46	1.16 (0.69-1.95)	.57		1.17 (0.70-1.96)	.56
**Sex**								
	Male (Ref.)							
	Female	1.05 (0.78-1.42)	.76	1.05 (0.77-1.41)	.77		1.05 (0.78-1.42)	.76
Age		1.00 (0.99-1.01)	.60	0.99 (0.98-1.00)	.18		0.99 (0.98-1.00)	.15
**Education**								
	Less than or high school (Ref.)							
	College or higher	1.45 (0.97-2.16)	.072	1.51 (1.01-2.67)	.045		1.53 (1.02-2.30)	.04
Full Model Wald *χ*^2^		45.61	<.001	54.26	<.001		55.05	<.001

^a^OR: odds ratio.

## Discussion

### Principal Findings

The key findings of this study included the characteristics of the users of mobile health technology across the life span ranging from 18 to 92 years. In this study, the likelihood of seeking health care support on the Web increased when adults had any or multiple chronic health conditions. A higher level of income and education than the general population was found to be related to the use of mobile health technology among adults. This is consistent with previous survey research that found high annual income was positively associated with higher usage of Internet technology [21]. We found that annual income of less than US $30,000 negatively impacted the use of mobile health technology. Annual income of above US $30,000 was positively associated with the use of mobile technology to seek Web-based support from a health care provider. Notably, our bivariate analysis showed a significant difference in the use of Web-based support by age. However, when adjusting for other demographic covariates and chronic conditions, age was no longer a considerable factor.

The findings of this study indicate that factors other than age have a greater impact on health support seeking from a provider facilitated by mobile technology. A randomized trial that supported Web-based data sharing between individuals with diabetes and health care providers resulted in a greater decrease in HbA1c compared with usual care over 6 months [22] and 1 year [23]. Mobile technology facilitated this interaction longitudinally. Adults across the age continuum with chronic health conditions, who receive health care provider support on the Web, will benefit from self-management of health facilitated by the technology. Mobile technology moves health care into the context of the individual’s daily life, filling gaps between episodic clinic visits.

A previous investigation found that many participants in the over 50-year age group reported a dislike of sharing health information on the Web through social media sites (eg, Facebook and Twitter) [24]. Middle-aged and older adults may perceive privacy as an issue in Web-based support seeking regarding their health. The survey used in this study did not address social media; however, these findings point to multiple considerations for promoting mobile health technology, which is still used less frequently than traditional offline support seeking for health issues.

The gap in mobile technology use of the lower socioeconomic group necessitates health care provider support to improve access to and use of mobile devices for the self-management of health. Integration with traditional health care practices involving in-person visits and phone calls must be considered. Health care providers can support individuals in self-management of their health by directing them to mobile technologies that are most effective [25], and to devices with optimal design to engage greater proportions of the population [26]. Health care provider support appears essential but is often overlooked for mobile health technology adoption among adults across the age continuum.

### Strengths and Limitations

One of the strengths of this study is the secondary analysis of a dataset collected to study mobile health technology. This study used a random sample of mobile phone users across the United States. In addition, the study was conducted in both English and Spanish to facilitate participation of the rapidly growing Hispanic population. A limitation of this study was the use of self-reported chronic conditions. This is a minor limitation, considering that no particular disease was targeted in this study. The dataset did not include information to delineate the type of health care support being sought, either general support or support for a chronic condition. Secondary analyses of existing data are limited to the existing variables in the dataset for investigation, and thus, unmeasured factors are a limitation. We examined sociodemographic variables and chronic condition variables that based on the literature were considered to be important in seeking health care support from providers on the Web. We acknowledge that the selected variables may not be sufficient to account for unmeasured factors related to our outcome variable. Other variables that may affect data and information exchange between providers and patients, such as depression and cognition, will be important to investigate in a future prospective study.

### Conclusions

This is one of the first studies to report the characteristics of adults seeking Web-based support from a health care provider facilitated by mobile technology. Overall, the findings reveal that adults of all ages use mobile technology. Multiple opportunities exist for health care providers to promote self-management of health facilitated by Internet-accessible technology. Many individuals continue to seek support from health care providers by conventional means; however, mobile health technology has considerable potential to improve traditional health care by extending the reach to at-risk groups and filling the gaps between episodic clinic visits. The evolving technology has the capability to provide individualized programs that effectively meet the needs of the individual. Future study is needed to better understand the disparity in Web-based support seeking for health issues, as well as the clinicians’ role in promoting access to and use of this technology to address the gaps identified in this study.

## Abbreviations

OR: odds ratio
SD: standard deviation
